# HS1,2 Ig Enhancer Alleles Association to AIDS Progression in a Pediatric Cohort Infected with a Monophyletic HIV-Strain

**DOI:** 10.1155/2014/637523

**Published:** 2014-05-20

**Authors:** Carla Montesano, Vincenzo Giambra, Domenico Frezza, Paolo Palma, Eliseo Serone, Guido Castelli Gattinara, Maurizio Mattei, Giorgio Mancino, Vittorio Colizzi, Massimo Amicosante

**Affiliations:** ^1^Department of Biology, University of Rome “Tor Vergata,” 00133 Rome, Italy; ^2^Terry Fox Laboratory, British Columbia Cancer Agency, Vancouver, BC, Canada V5Z 1L3; ^3^University Department of Pediatrics, DPUO, Unit of Immune and Infectious Diseases, Children's Hospital “Bambino Gesù,” 00165 Rome, Italy; ^4^Istituto Mario Negri Sud, 66030 Chieti, Italy; ^5^International Training Unit, Children's Hospital “Bambino Gesù,” 00165 Rome, Italy; ^6^San Pietro Fatebenefratelli Hospital, 00189 Rome, Italy; ^7^Department of Biomedicine and Prevention, University of Rome “Tor Vergata,” 00133 Rome, Italy; ^8^ProxAgen Ltd., 1505 Sofia, Bulgaria

## Abstract

Alteration in the humoral immune response has been observed during HIV infection. The polymorphisms of enhancer HS1,2, member of the 3^′^ regulatory region of the Ig heavy chain cluster, may play a role in the variation of the humoral response leading to pathological conditions. To assess the role of the HS1,2 polymorphic variants in the progression of AIDS, the HS1,2-A allelic frequencies were investigated in a cohort of HIV infected pediatric subjects from a nosocomial outbreak with a monophyletic strain of HIV. From a total group of 418 HIV infected children in the outbreak cohort, 42 nonprogressors and 31 progressors without bias due to antiretroviral therapy were evaluated. HS1,2 allele ^∗^1 has been associated with nonprogressors (allelic frequency: 51.19% versus 33.87% in progressors, OR 0.5, and *P* = 0.0437), while allele ^∗^2 has been associated with progression (allelic frequency: 48.39% versus 30.95% in nonprogressors, OR 2.1, and *P* = 0.0393). Further, only subjects carrying allele ^∗^2 in absence of allele ^∗^1, either in homozygous condition for allele ^∗^2 [nonprogressors 2/42 (4.76%), Progressors 7/31 (22.58%), OR 5.8, and *P* = 0.0315] or in combination with other allelic variants [nonprogressors 7/42 (16.67%), Progressors 13/31 (41.93%), OR 3.61, and *P* = 0.0321], have been associated with HIV progression to AIDS. In conclusion, while the HS1,2 allele ^∗^1 has a protective effect on HIV progression when present, allele ^∗^2 is associated with progression toward AIDS when allele ^∗^1 is absent.

## 1. Introduction

In HIV infected subjects, the progression to AIDS has been associated with a number of host genetic factors [1] and virological variants [2], suggesting that the individual immune-response to HIV might be influenced at many levels of the virus-host interaction.

The principal targets of HIV are the CD4+ T-lymphocytes whose decline causes the impairment of the host immune defences [3]. However, alteration in the humoral immune-response mediated by B-cells, including hypergammaglobulinemia [4, 5], has been observed during HIV infection in children and adults [6, 7] particularly in the late stages of disease.

The humoral response is the result of interactions whose modulation is partially depending on polymorphic features of genes leading to Ig maturation and production. The 3′Ig heavy chain regulatory regions mainly control Ig heavy chain expression [8].

**Figure 1:**
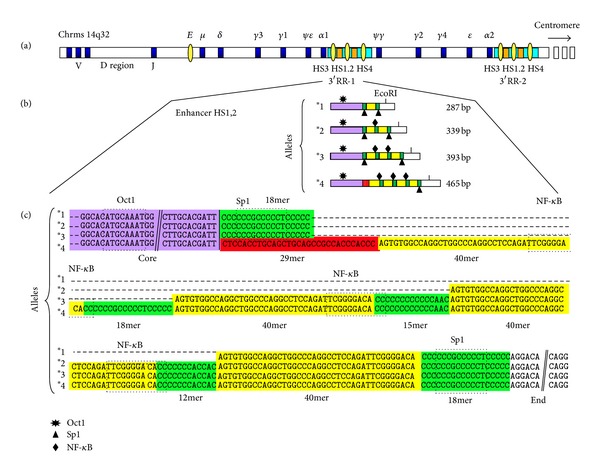
Schematic map of the constant heavy chain region: (a) location of the two regulatory regions within the heavy chain genes on chromosome 14q32; (b) enlargement of the 3′RR-1 hs1.2 polymorphic enhancer and schematic representation of the four alleles; (c) nucleotidic sequence of the hs1.2 enhancer with the differences of the four alleles and the consensus for the Sp1 and NF-kB transcription factor consensus.

The 3′ regulatory region at the 3′ of the Ig *α*-1 constant gene includes three enhancers among which only the central HS1,2-A is polymorphic (see Figure 1) [9]. This region is involved in B lymphocytes maturation and Ig production [10]. Furthermore, the different allelic variants have been associated with a different capability of controlling Ig levels [11].

The HS1,2-A alleles have a remarkable variability in frequency among world populations [12] and the allele *2 variant of the HS1,2-A has been associated with susceptibility to several immune-related diseases [13–16], suggesting that it might contribute to pathological conditions.

A progressive impairment of B cell response to recall antigens, the increased polyclonal activation of B cells, and the expression of activation markers on their surface have been observed in HIV patients [4–7]. Because no specific B cell-related factors have been associated with HIV protection, in this study we investigated the frequency of the Ig 3′ enhancer HS1,2-A alleles in the progression toward AIDS. To minimize the influence of genetic background (due to a population originating from different or large geographic areas) and viral strain variability on HIV progression, we focused the analysis on a cohort of HIV infected subjects from a nosocomial outbreak with a monophyletic strain of HIV in the Benghazi Children Hospital in Libya [17].

## 2. Patients and Methods

### 2.1. Study Population

The cohort involved in the outbreak of HIV infection, at the “El-Fath Children's Hospital” of Benghazi, includes 418 children, 18 mothers, and 2 nurses. All the children were infected after hospitalization or after attending the hospital outpatients'. The HIV infection, outbreak, immunological, and immunogenetics characterizations of the cohort have been previously described [17, 20–22], indicating in the cohort the presence of a defined cluster of HIV-1 clade A/G virus (CRF02_AG).

HS1,2-A typing was performed upon residual blood sample availability, after informed consent [21, 22], from a subgroup of 73 children. According to CDC classification and the literature [18, 19], patients were divided into nonprogressors (*N* = 42) and progressors (*N* = 31) toward AIDS.  Table 1 summarizes the demographic and clinical characteristic of patients.

The cohort subgroup evaluated in this study does not differ substantially from the entire cohort for the mean value of CD4 cell counts, the viral load, and the classification groups of AIDS progression. Thus, as far as the infection is concerned, the patients can be considered a homogeneous pool of subjects.

### 2.2. DNA Extraction

DNA was extracted from the serum samples. Briefly, 300 microliters of serum was treated with proteinase K and phenol-chloroform by standard procedure. The supernatant was then reextracted with chloroform/isoamilic alcohol and processed by the “microcon 100” method (Millipore, Bedford MA, USA).

### 2.3. PCR Analysis and Genotyping

The PCR amplifications were performed with 50 nanograms of genomic DNA as described by Giambra et al. [23]. The two nested PCRs discriminate the two HS1,2-A and -B enhancers present in the regulatory regions at the 3′ of both constant *α*-1 and *α*-2 genes and the different alleles were analysed by gel agarose 2.5% electrophoresis [23].

### 2.4. Statistical Analysis

Allelic, phenotypic, and genotypic frequencies data are expressed as percentages with odds ratio (OR) when appropriate. Comparisons between frequencies in the study groups have been performed by Fisher's exact test. Hardy-Weinberg equilibrium has been evaluated as previously described [12]. All the statistical analyses have been carried out with GraphPad Prism (GraphPad Software Inc., San Diego, CA) packages.

## 3. Results and Discussion

In HIV infection, host genetic factors [1, 21, 22, 24–27], mostly related to the impairment of T cell response or with the increase of an inflammatory status, have been associated with AIDS progression. Differently, no specific factors have been identified or associated with the progression of AIDS in terms of production of antibodies, although alteration in the humoral immune response, such as progressive impairment of response to recall antigens and hypergammaglobulinemia during late stage of HIV infection, has been observed [4–6], where Ig 3′ enhancer HS1,2-A might play a role.

The observed frequencies for the HS1,2 genotypes in the overall population are in agreement with Hardy-Weinberg equilibrium, (*P* > 0.9) suggesting that the population is homogeneous. Further, the HS1,2 allelic frequency distribution of this Libyan population coming from the area of Benghazi is not different from other Mediterranean populations (data not shown, [12]).

Table 2 shows the allelic frequency analysis in the study groups. A statistically significant increase of allele *1 frequency has been observed in HIV nonprogressors (51.19%, versus 33.87% in progressors, *P* = 0.0437), while allele *2 has been found to increase in progressors (48.39%, versus 30.95% in nonprogressors, *P* = 0.0393).

This observation is further supported by the genotypic analysis (Table 3), where a statistically significant increase of the 2/2 genotype in HIV progressor subjects has been observed [nonprogressors 2/42 (4.76%), progressors 7/31 (22.58%), *P* = 0.0315].

Finally, the frequency of subjects carrying allele *1 resulted increased in nonprogressors (80.95% versus 58.06% in progressors, *P* = 0.0398) (Table 4), whereas the frequency of subjects carrying allele *2 did not differ between the two patient groups (Table 4), either for the small population size evaluated or for a potential protective effect of allele *1. In fact, subjects carrying allele *2, in the absence of allele *1, resulted in significant increase in progressors (13/31, 41.93%) rather than nonprogressors (7/42, 16.67%; OR 3.61, *P* = 0.0321) (see also Table 3).

We have already observed in other immune pathologies a significant increase of the frequency of the enhancer HS1,2 allele *2 [13, 14], supporting the hypothesis that the specific allelic conformations might trigger the different immune response leading to the hypergammaglobulinemia or other critical responses to cell-cell interaction and signalling. This hypothesis was confirmed in selective IgA deficiency where HS1,2 polymorphism influences Ig serum production [15]. This is consistent with both* in silico* analysis and EMSA experiments demonstrating the presence of a consensus binding site for NF-kB in allele *2 but not in allele *1 [11, 25, 28].

Consequently, the allele *2, capable of recruiting NF-kB, might give a different efficiency to the 3′ regulatory region towards Ig transcription. Interestingly NF-kB through the consensus on promoters of Bruton tyrosine kinase (Btk) indirectly regulates also the B cells' activating factor (BAFF) and the B cells' survival in response to BCR activation [29]. Likewise higher level of plasma BAFF has been associated with progression status and hypergammaglobulinemia in HIV infected patients [30].

Moreover, the presence of NF-kB in allele *2 might also be responsible of B cell activation by cytokines such as TNF*α*, IL-6, IFN*α* produced in HIV viremic patients [6]. In turn the B cells activated by inflammatory stimuli might themselves induce activation of other immune cells and contribute to HIV progression.

Alternatively, the presence (yet to be identified) of specific haplotypes, linked to allele *2, might be at the basis of the progression to AIDS which is observed in subjects homozygous for allele *2.

In conclusion, despite possible limitations related to the size of our population sample, this study sheds new light on the relevance of new genetic factors involved in B cell exhaustion, associated with progression of AIDS in children. The application of the model developed in this study to a larger population might contribute to further clarification of the mechanisms behind the association of genetic susceptibility of the HS1,2 with HIV-1 and to the definition of the role of this marker in the early identification of subjects with HIV infection more prone to progression to AIDS, ensuring a proper followup and treatment.

## Figures and Tables

**Table 1:** Demographic and clinical characteristics of the study groups classified according to clinical progression [18, 19].

	Patients	Sex (% male)	Median age at the first observation (IQR^1^)	Median Nadir %CD4+ (IQR^1^)	HIV viral load median (range)
Nonprogressors	42	57.10%	5.47 (1.80–10.04)	26.1% (24.0–31.0)	650 (50–17000)
Progressors	31	58.06%	3.88 (2.18–6.55)	15.1% (11–18)	73000 (5280–500000)

^1^IQR: interquartile range.

**Table 2:** Allelic frequency of the HS1,2-A alleles in HIV-infected nonprogressor and progressor patients.

Allele	Nonprogressors (84 alleles)		Progressors (62 alleles)		O.R.	*P* (Fisher's exact test)
	*N *	Frequency	*N *	Frequency		
1	43	0.5119	21	0.3387	0.4884	**0.0437**
2	26	0.3095	30	0.4839	2.0910	**0.0393**
3	6	0.0714	4	0.0645	0.8966	1
4	9	0.1071	7	0.1129	1.0610	1

In bold significant *P* values.

**Table 3:** Frequency of the HS1,2-A genotypes in HIV-infected nonprogressor and progressor patients.

Genotypes	Nonprogressors *N* = 42		Progressors *N* = 31		O.R.^1^	*P* (Fisher's exact test)^1^
	*N *	Frequency	*N *	Frequency		
1*∖*1	9	0.2143	3	0.0968	0.3929	0.2169
1*∖*2	17	0.4048	10	0.3226	0.7003	0.6244
1*∖*3	3	0.0714	2	0.0645	0.8966	1
1*∖*4	5	0.1190	3	0.0968	0.7929	1
2*∖*2	2	0.0476	7	0.2258	5.8333	**0.0315**
2*∖*3	2	0.0476	2	0.0645	1.3793	1
2*∖*4	3	0.0714	4	0.1290	1.9259	0.4484
3*∖*3	0	0	0	0	NA	NA
3*∖*4	1	0.0238	0	0	NA	1
4*∖*4	0	0	0	0	NA	NA

^1^NA: not applicable.

In bold significant *P*-values.

**Table 4:** Frequency of subjects carrying at least one copy of the HS1,2-A alleles in HIV-infected nonprogressor and progressor patients.

Allele	Nonprogressors *N* = 42		Progressors *N* = 31		O.R.	*P* (Fisher's exact test)
	*N *	Frequency	*N *	Frequency		
1	34	0.8095	18	0.5806	0.3258	**0.0398**
2	24	0.5714	23	0.7419	2.1562	0.1477
3	6	0.1429	4	0.1290	0.8889	1
4	9	0.2143	7	0.2258	1.0694	1

In bold significant *P* values.
